# CaTFormer: Prior-Guided Transformer with Hierarchical Supervision for Baggage Re-Identification

**DOI:** 10.3390/s26175409

**Published:** 2026-08-27

**Authors:** Aixing Li, Chunwei Zheng, Li Zhang, Ke Gong

**Affiliations:** 1 School of Information Science and Engineering, Chongqing Jiaotong University, Chongqing 400074, China; aixing.li@cqjtu.edu.cn (A.L.); zhengchunwei2025@163.com (C.Z.); 2 School of Digital Economy and Management, Chongqing Polytechnic University of Electronic Technology, Chongqing 401331, China; elisazl@163.com; 3 School of Economics and Management, Chongqing Jiaotong University, Chongqing 400074, China

**Keywords:** baggage re-identification, prior-guided transformer, camera-aware embedding, textual-attribute embedding, hierarchical supervision

## Abstract

Baggage re-identification (ReID) aims to retrieve the same baggage instance across non-overlapping cameras or viewpoints, providing a practical tool for intelligent baggage management in structured transportation surveillance scenarios. Compared with pedestrian and vehicle ReID, baggage ReID is more challenging because baggage items often exhibit homogeneous appearances, limited semantic structures, and subtle identity cues that are sensitive to viewpoint, illumination, blur, and background changes. To address these challenges, we propose CaTFormer, a prior-guided transformer framework for baggage ReID. CaTFormer incorporates camera-aware and textual-attribute embeddings into Swin-based visual representation learning. An Adaptive Multi-Embedding Fusion (AMEF) module coordinates camera, color, and type priors before injecting the fused prior representation into visual tokens. In addition, a Candidate-Selected Semantic-Dominant Hierarchical Loss (CS-HL) supervises multi-level Swin features using a fixed stage-weight vector selected from a constrained candidate set. Experiments on the self-collected BaggageID dataset show that CaTFormer achieves 62.0% mAP, 85.4% Rank-1 accuracy, and 94.0% Rank-5 accuracy, outperforming the compared open-source ReID models under the same protocol. On the public MVB benchmark, CaTFormer obtains 87.0% mAP and 87.6% Rank-1 accuracy. Additional evaluations provide supplementary evidence for the applicability of CaTFormer and its CS-HL-based reduced variant in broader ReID settings.

## 1. Introduction

Checked baggage handling is a highly structured transportation process in which baggage items are transported through multiple belts, transfer points, and inspection areas before being delivered to passengers or downstream logistics systems. In such environments, visual traceability is important for locating delayed or mishandled baggage, supporting anomaly inspection, and improving baggage management efficiency. Baggage re-identification (ReID), which retrieves the same baggage instance across non-overlapping cameras or viewpoints, provides a practical visual retrieval tool for this structured surveillance scenario.

ReID has been widely studied in pedestrian and vehicle retrieval. Representative studies have explored discriminative training objectives [1], multi-granularity feature learning [2], and transformer-based retrieval architectures [3]. These studies provide important methodological foundations for learning discriminative ReID representations. However, baggage ReID has its own representation difficulty. Baggage items often have simple geometric shapes, limited semantic parts, and highly similar appearances. Many instances differ only in subtle cues, such as color shade, surface texture, stickers, scratches, handle shape, or local wear. These cues are much weaker than human body structure or vehicle-specific components, making identity discrimination highly dependent on fine-grained visual evidence.

These fine-grained cues are also condition-sensitive. In airport baggage handling systems, the same item may be captured by cameras installed at different positions, with different illumination, motion blur, resolution, and background clutter. As a result, the reliability of visual attributes may vary across cameras and viewpoints. For example, color may be informative under stable illumination but unreliable under strong lighting variation, while local texture may be weakened by low resolution or motion blur. Prior-guided ReID studies have shown that attribute cues [4], viewpoint information [5], and camera-related embeddings [3] can complement visual features in retrieval tasks. In structured baggage surveillance, camera context and simple baggage attributes can therefore serve as useful priors, but their contribution may vary with acquisition conditions.

Existing baggage ReID studies have mainly explored similarity-based matching [6], local or material-aware feature learning [7,8], and multi-view or camera-aware modeling [9,10]. These methods have advanced baggage-oriented retrieval from different perspectives. Nevertheless, existing baggage ReID studies have not fully examined how structured camera and attribute information can be jointly integrated with hierarchical visual representations for baggage retrieval.

In practical airport surveillance systems, camera identifiers are naturally available from fixed camera networks, and basic baggage attributes, such as color and type, can often be obtained from structured records, manual query input, or lightweight annotation. This work therefore focuses on baggage ReID in structured transportation surveillance scenarios, where visual images may be associated with such auxiliary priors. The key challenge is not merely to add these priors to the visual backbone, but to coordinate them according to their condition-dependent reliability.

To address this challenge, we propose CaTFormer (Camera- and Textual-Attribute Guided Transformer), a baggage-oriented transformer framework based on a Swin backbone. CaTFormer integrates camera-aware and textual-attribute embeddings into visual representation learning through an Adaptive Multi-Embedding Fusion (AMEF) module. AMEF coordinates heterogeneous priors using channel-wise attention and learnable branch weighting before injecting the fused prior representation into the visual tokens. In addition, we introduce a Candidate-Selected Semantic-Dominant Hierarchical Loss (CS-HL), which supervises multi-level Swin features using a fixed stage-weight vector selected from a constrained semantic-dominant candidate set.

The main contributions of this study are summarized as follows:

- We propose CaTFormer, a baggage-oriented prior-guided transformer framework for structured transportation surveillance scenarios. The framework uses an AMEF module to coordinate camera, color, and type priors before injecting the fused prior representation into Swin-based visual token learning.
- We introduce CS-HL for multi-level feature supervision, which selects a fixed stage-weight vector from a semantic-dominant candidate set and uses the selected weights for full training.
- Experiments on the self-collected BaggageID dataset and the public MVB benchmark demonstrate the effectiveness of CaTFormer for baggage ReID. CaTFormer achieves better results than the compared open-source ReID models on BaggageID and competitive retrieval performance in the MVB comparison. Supplementary evaluations on additional public ReID datasets further examine the applicability of the proposed design.

The remainder of this paper is organized as follows. Section 2 reviews related work. Section 3 describes the proposed method. Section 4 presents the experimental results and analysis. Section 5 concludes the paper.

## 2. Related Work

### 2.1. Representation Learning Objectives for ReID

Re-identification (ReID) aims to associate object instances across non-overlapping cameras or acquisition conditions. Supervised ReID commonly learns discriminative image embeddings through identity classification and metric learning objectives. Ahmed et al. [11] used pairwise verification losses to determine whether two images belong to the same identity, while Zheng et al. [12] treated each training identity as a separate class and optimized the embedding through cross-entropy supervision. FaceNet [13] formalized triplet-based metric learning by pulling positive samples closer while pushing negative samples apart in the embedding space. The strong baseline proposed by Luo et al. [1] further showed that combining identity classification with metric learning losses can produce effective retrieval representations. These studies provide the basic optimization framework for supervised ReID representation learning.

Together, these objectives define the dominant supervised learning paradigm in ReID, where classification losses encourage inter-class separability and metric learning losses constrain the relative distances among samples in the embedding space. This paradigm also provides a common training basis for baggage ReID methods.

### 2.2. Transformer-Based and Hierarchical ReID Models

Transformer architectures have recently been adopted in ReID because self-attention can model long-range dependencies and token interactions. TransReID [3] is a representative transformer-based ReID framework, DC-Former [14] explores compact transformer design for person ReID, and Wang et al. [15] combine CNN and transformer features to enhance retrieval representation. These studies indicate that transformer-based architectures provide a useful design direction for ReID representation learning across different object categories.

In addition to global token modeling, hierarchical or multi-level feature representation has been explored in different ReID tasks. HAT [16] integrates hierarchical transformer features for person retrieval, MFAT [17] explores multi-level feature aggregation in transformer-based ReID, and Pyramidal Transformer [18] indicates the value of combining hierarchical token representations for retrieval. Swin Transformer [19] is a general vision backbone that produces multi-scale feature maps through shifted-window attention, making it suitable for modeling both local detail and global context. For baggage ReID, such hierarchical features are particularly relevant because shallow stages can preserve fine-grained appearance cues, whereas deeper stages capture more stable semantic information. These studies suggest the value of hierarchical features, but the allocation of supervision across different feature levels remains less explicitly studied in baggage ReID.

### 2.3. Fine-Grained and Prior-Guided Representation Learning

Fine-grained feature modeling is critical when different identities have similar global appearances. Relation Network [20] strengthens local matching between pedestrian images, MGN [2] combines global and local features for person ReID, and the two-level attention network proposed by Guo et al. [21] explores multi-grain representation learning for vehicle ReID. In transformer-based ReID, TransReID [3] uses jigsaw patch learning to strengthen local patch interaction, Mishra et al. [22] introduce part-level tokenization for occluded person ReID, and AAFormer [23] improves local correspondence through auto-aligned transformer modeling. These methods indicate that retrieving highly similar identities often requires representations that retain local details rather than relying only on global descriptors.

Beyond visual details, prior-guided ReID methods use semantic attributes and acquisition conditions in different forms. AttributeNet [4] and viewpoint-aware metric learning [5] incorporate attribute or viewpoint supervision, while Lian et al. [24] combine a color/type side branch with global and transformer-based attention branches. MsKAT [25] represents camera and viewpoint information as state knowledge and color and type as attribute knowledge, and uses State Elimination and Attribute Aggregation Transformers to interact the corresponding knowledge vectors with multi-scale visual features.

Other methods construct explicit attribute representations and then learn their aggregation. MAAT [26] encodes color, model, and viewpoint as learnable embeddings, processes their concatenation through convolution and channel attention, and adds the aggregated attribute embedding to each image patch token. Tumrani and Siddiqui [27] derive a color descriptor from an HSV histogram and a type descriptor from a lightweight CNN, and then use a channel-wise gate to fuse the combined attribute vector with the global Swin descriptor. These designs provide relevant context for baggage ReID, where camera identity supplies acquisition context and color and type attributes provide semantic cues whose reliability can vary with illumination, viewpoint, blur, and resolution.

### 2.4. Baggage Re-Identification Methods

Baggage ReID has received increasing attention as a retrieval task for airport surveillance and baggage handling. Early studies commonly formulated the task as pairwise verification. Zhang et al. [28] introduced the MVB dataset and a merged Siamese network for baggage ReID, while Mazzeo et al. [6] used pretrained CNN backbones within a Siamese framework for non-invasive baggage re-identification. These works established the feasibility of visual matching for baggage retrieval.

Subsequent studies further addressed baggage-specific appearance and acquisition challenges. AttentionNet [29], SMBNet [7], and LSDNN [8] place additional emphasis on foreground or local regions. AttentionNet uses weak region annotations to guide foreground attention in few-shot retrieval, whereas SMBNet and LSDNN combine global and local representations and incorporate material information into feature learning. RGViT [30] performs region-to-global feature modeling in a Transformer architecture through regional self-attention and global spatial-relation attention, together with quadruplet supervision. MVAD-Net [31] and QuadNet [9] both explicitly model multi-view variation; MVAD-Net combines multi-view attention with domain-invariant learning, whereas QuadNet addresses view variation at the sample, feature, and loss levels. CDR-CARNet [10] further combines cross-domain robust feature learning with dynamic hard-sample mining and camera-aware re-ranking. These studies examine baggage ReID from multiple perspectives involving local appearance, material attributes, region-to-global representation, view variation, domain discrepancy, and camera information, providing the context for integrating structured priors with multi-level visual representations.

## 3. Methodology

**Figure 1:**
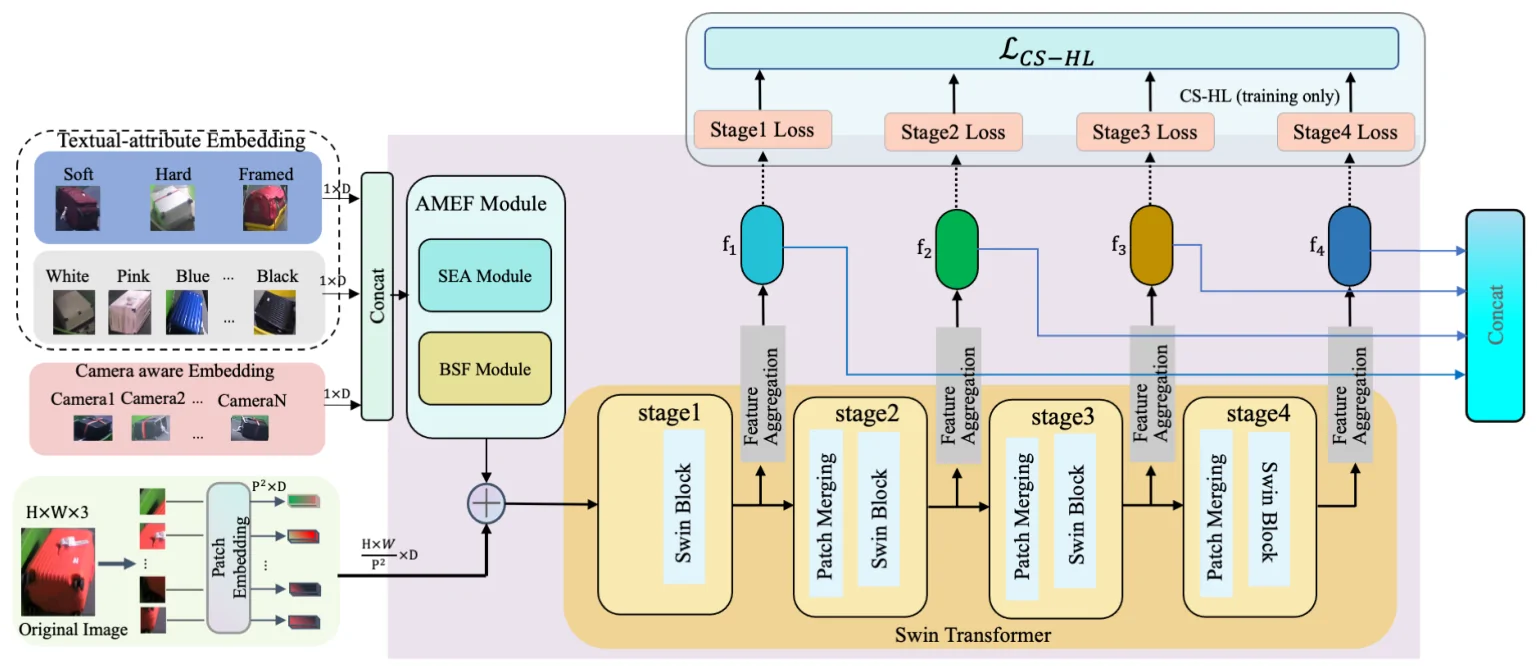
Framework of the proposed CaTFormer. “⊕” denotes broadcasting addition of the fused prior embedding to the image patch tokens. CS-HL and the four stage-wise loss branches are used only during training.

The proposed baggage ReID framework is built on the hierarchical Swin transformer. As illustrated in Figure 1, the model encodes camera identity with a camera-aware embedding (CaE) and encodes baggage color and type with textual-attribute embeddings (TaE). The adaptive multi-embedding fusion (AMEF) module, which contains a squeeze-and-excitation attention (SEA) unit and a branch soft attention fusion (BSF) unit, fuses these three prior embeddings; the fused embedding is then added to the image patch tokens before Swin feature extraction. The four Swin stages produce multi-level representations. During training, Candidate-Selected Semantic-Dominant Hierarchical Loss (CS-HL) supervises the stage-wise features; during inference, the concatenated stage-wise features form the retrieval descriptor. Section 3.1, Section 3.2, Section 3.3, Section 3.4 and Section 3.5 describe these components in detail.

### 3.1. Basic Swin Transformer

We employed the Swin transformer as the baseline model for baggage ReID. As depicted in Figure 1, it partitions an RGB image of size H×W into $N_{p}$ nonoverlapping patches of size P×P, where $N_{p}=HW/P^{2}$. The *n*-th patch is denoted by $x_{p}^{n}\in R^{P\times P\times 3}$, where 3 represents the number of image channels. Subsequently, each patch is passed through a linear embedding layer, which projects raw pixel values of size P×P×3 into a *D*-dimensional feature space, yielding the input sequence

(1) $$Z_{0}=\left[F\left(x_{p}^{1}\right);F\left(x_{p}^{2}\right);\ldots ;F\left(x_{p}^{N_{p}}\right)\right],$$

where F(·) denotes linear projection. After mapping, we obtain $Z_{0}\in R^{\frac{H}{P}\times \frac{W}{P}\times D}$.

The Swin transformer employs a four-stage hierarchical architecture, where each stage progressively merges image patches to generate feature maps with decreasing spatial resolution and increasing channel dimension, thereby achieving multiscale feature representation. Each stage consists of a patch-merging downsampling layer, followed by several Swin transformer blocks. Within each block, shifted-window multi-head self-attention and a multilayer perceptron are applied, along with residual connections and layer normalization. At the *k*-th stage, the output feature map, $Z_{k}$, exhibits a size of

$$\frac{H}{2^{k-1}P}\times \frac{W}{2^{k-1}P}\times 2^{k-1}D.$$

In the baseline model, the feature map output from the final stage is adopted as the representation for baggage ReID.

**Supervised Learning.** In the baseline, the network is optimized using identity classification loss (ID loss) [12] and triplet loss [13]. ID loss employs the following cross-entropy loss:

(2) $$L_{ID}=-\sum_{m=1}^{C}y_{m}\log\left({\hat{y}}_{m}\right),$$

where *C* denotes the number of identity classes in the training set, $y_{m}$ denotes the ground-truth indicator of class *m*, and ${\hat{y}}_{m}$ denotes the predicted probability of class *m*.

Triplet loss is employed to further enhance discriminative feature learning. Given a triplet {a,p,n}, where *a* denotes the anchor, *p* denotes a positive sample of the same identity, and *n* denotes a negative sample of a different identity, the objective is to minimize the intraclass distance while maximizing the interclass distance. We employed the following noise-robust, soft-margin triplet loss [1]:

(3) $$L_{T}=\log\left(1+\exp\left({∥f_{a}-f_{p}∥}_{2}^{2}-{∥f_{a}-f_{n}∥}_{2}^{2}\right)\right),$$

where ${∥f_{a}-f_{p}∥}_{2}^{2}$ and ${∥f_{a}-f_{n}∥}_{2}^{2}$ represent the squared Euclidean distances between the anchor and positive feature vector and between the anchor and negative feature vector, respectively.

### 3.2. Camera-Aware Embedding

Cross-camera variation is a common source of appearance shift in structured baggage surveillance. To encode this acquisition context, we introduce a camera-aware embedding (CaE) at the input token level.

Let $E_{cid}\in R^{N_{cid}\times D}$ denote the camera embedding matrix, where $N_{cid}$ is the number of cameras and *D* is the token dimension. For an image captured by the *v*-th camera, the corresponding camera prior is obtained by lookup:

(4) $$e_{cid}=E_{cid}\left[v\right],\;e_{cid}\in R^{1\times D}.$$

The same camera embedding is shared by all patch tokens from the image and serves as one of the structured priors used in the subsequent fusion module.

### 3.3. Textual-Attribute Embedding

Simple baggage attributes provide complementary semantic cues when visual differences are subtle. In this work, baggage type and color are encoded as textual-attribute embeddings (TaE) and used as structured priors for baggage ReID.

Let $E_{typ}\in R^{N_{typ}\times D}$ and $E_{clr}\in R^{N_{clr}\times D}$ denote the learnable embedding matrices for baggage type and color, respectively. For an image with type label $l_{typ}$ and color label $l_{clr}$, the corresponding attribute priors are

(5) $$e_{typ}=E_{typ}\left[l_{typ}\right],\;e_{clr}=E_{clr}\left[l_{clr}\right],\;e_{typ},e_{clr}\in R^{1\times D}.$$

The resulting type and color embeddings are learned jointly with the visual backbone and serve as the remaining structured priors for adaptive multi-embedding fusion.

### 3.4. Adaptive Multi-Embedding Fusion Module

**Figure 2:**
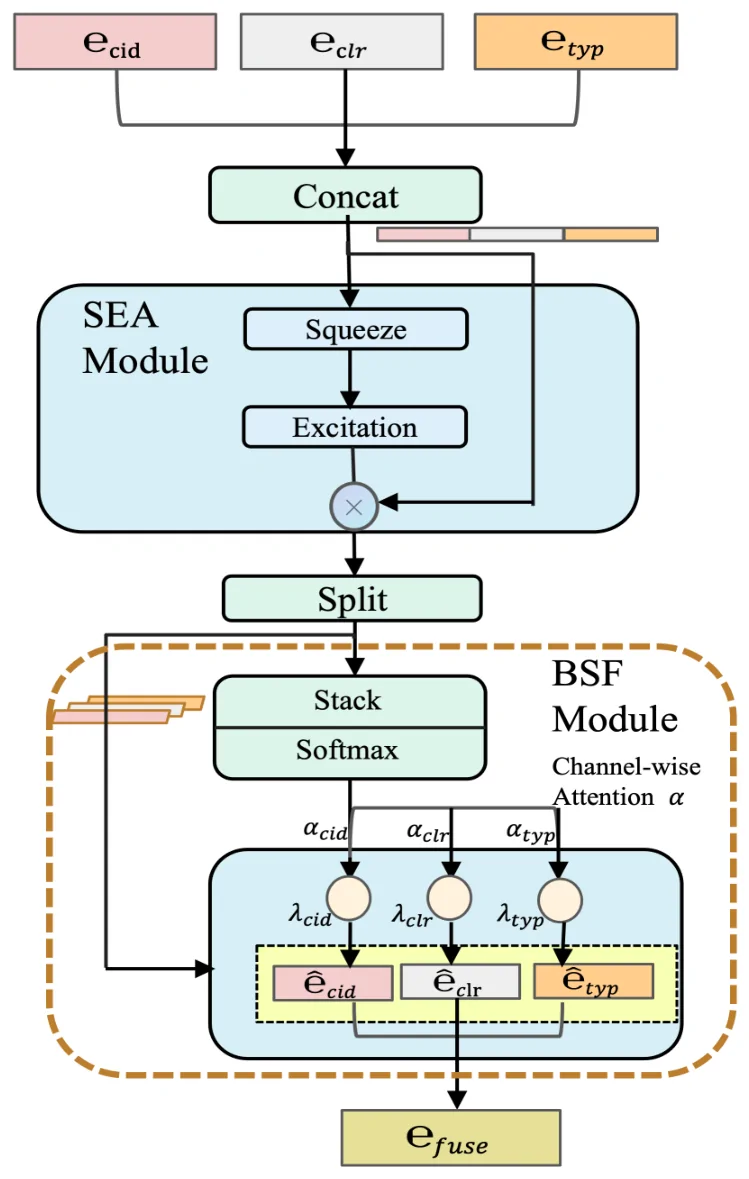
Structure of the AMEF module.

Different priors may contribute unequally under different visual conditions. As illustrated in Figure 2, AMEF coordinates the camera, color, and type embeddings using a two-level fusion design: SEA reweights the concatenated prior channels, whereas BSF combines channel-wise branch attention with learnable branch-level coefficients before the fused prior is injected into the visual tokens. The pink, gray, and orange paths in the figure trace the camera, color, and type embeddings, respectively.

Throughout Section 3, bold uppercase symbols denote matrices or token tensors, bold lowercase symbols denote vectors or image-patch tensors, and italic symbols denote scalars, indices, labels, or individual vector elements. The three prior embeddings are first concatenated along the channel dimension:

(6) $$e_{cat}=\mathrm{Concat}\left(e_{cid},e_{clr},e_{typ}\right),\;e_{cat}\in R^{1\times 3D}.$$

The SEA unit [32] reweights the concatenated representation along the channel dimension and produces $e_{\mathrm{SEA}}$ with the same dimensionality as $e_{cat}$.

The BSF unit then splits $e_{\mathrm{SEA}}$ into three branch-specific embeddings, denoted as ${\hat{e}}_{cid},{\hat{e}}_{clr},{\hat{e}}_{typ}$. Let S={cid,clr,typ} denote the set of prior branches. The three branch-specific embeddings are stacked along a branch dimension, and for each channel position, a softmax is applied across this dimension:

(7) $$\alpha_{b}=\frac{\exp\left({\hat{e}}_{b}\right)}{\sum_{b^{\prime }\in S}\exp\left({\hat{e}}_{b^{\prime }}\right)},\;b\in S,$$

where $\alpha_{b}\in R^{1\times D}$ is the channel-wise attention vector for branch *b*.

In addition, learnable branch coefficients are used to model the global contribution of each prior:

(8) $$\lambda_{b}=\frac{\exp(\theta_{b})}{\sum_{b^{\prime }\in S}\exp\left(\theta_{b^{\prime }}\right)},\;b\in S,$$

where $\theta_{b}$ is the learnable scalar parameter of branch *b*, and $\lambda_{b}$ is the corresponding softmax-normalized global weight.

The final fused embedding is then computed by combining global branch weighting and channel-wise attention:

(9) $$e_{fuse}=\beta \sum_{b\in S}\lambda_{b}\left(\alpha_{b}⊙{\hat{e}}_{b}\right),$$

where β is a learnable fusion gain factor and ⊙ denotes element-wise multiplication. This formulation combines channel-level attention and branch-level weighting for adaptive prior fusion.

Finally, $e_{fuse}$ is broadcast along the token dimension and added to the projected patch tokens:

(10) $$Z_{0}^{\prime }=Z_{0}+e_{fuse}.$$

Thus, AMEF injects the fused camera, color, and type priors into the projected patch tokens before the four-stage Swin feature extraction.

### 3.5. Candidate-Selected Semantic-Dominant Hierarchical Loss

The Swin backbone provides four-stage hierarchical representations. In the baseline setting, only the final stage is directly supervised for identity discrimination. Let $f_{4}$ and $s_{4}$ denote the ReID feature and classification logits obtained from the fourth Swin stage, respectively. The baseline objective is defined as

(11) $$L_{base}=\kappa L_{ID}\left(s_{4},y\right)+\xi L_{T}\left(f_{4},y\right),$$

where *y* denotes the identity label, $L_{ID}$ is the identity classification loss, $L_{T}$ is the batch-wise triplet loss, and κ and ξ are the corresponding loss coefficients.

Although the final Swin stage contains stronger high-level semantics, lower stages can preserve fine-grained baggage cues, such as local texture, color details, stickers, and surface marks. Therefore, CS-HL applies ReID supervision to all Swin stages. For the *k*-th stage, the stage-wise loss is written as

(12) $$L_{k}=\kappa L_{ID}\left(s_{k},y\right)+\xi L_{T}\left(f_{k},y\right),\;k=1,\ldots ,K,$$

where K=4, and $f_{k}$ and $s_{k}$ denote the ReID feature and classification logits of stage *k*, respectively. The triplet term is computed over identity-balanced mini-batches, as described in Section 4.2.

The key issue is how to assign supervision weights to different stages. Manually fixed weights may not be optimal across baggage datasets with different image quality, viewpoint variation, and fine-grained ambiguity. Conversely, directly learning weights from mini-batch losses can be biased toward stages that provide stronger short-term optimization signals. To keep the supervision interpretable and stable, CS-HL selects a fixed stage-weight vector through a coarse-to-fine candidate screening strategy.

Let $b^{\left(r\right)}$ denote the supervision budget assigned to the final stage in the *r*-th candidate, and let $p^{\left(r\right)}=\left(p_{1}^{\left(r\right)},\ldots ,p_{K-1}^{\left(r\right)}\right)$ denote the allocation pattern for the first K−1 stages, where $\sum_{k=1}^{K-1}p_{k}^{\left(r\right)}=1$ and $p_{k}^{\left(r\right)}\geq 0$. The candidate weight vector is constructed as

(13) $$w^{\left(r\right)}=\left[\left(1-b^{\left(r\right)}\right)p_{1}^{\left(r\right)},\ldots ,\left(1-b^{\left(r\right)}\right)p_{K-1}^{\left(r\right)},b^{\left(r\right)}\right],\;b^{\left(r\right)}\in B,\;p^{\left(r\right)}\in P,$$

where B is a predefined semantic-budget set and P is a predefined auxiliary-allocation pattern set. By constraining $b^{\left(r\right)}>0.5$, the final stage remains the dominant semantic supervision source, while the remaining budget is distributed to lower stages to preserve local and middle-level cues. In practice, an initial coarse candidate set is first constructed from B and P. If several candidates show close screening performance, a local refinement candidate set is further generated around the leading candidates while preserving the semantic-dominant constraint.

The selected vector $w^{*}=\left[w_{1}^{*},\ldots ,w_{K}^{*}\right]$ is determined from the coarse and locally refined candidates using a held-out validation subset constructed exclusively from the official training identities; the official query and gallery sets are not used for candidate selection. Once selected, $w^{*}$ is fixed throughout full training. The final CS-HL objective is

(14) $$L_{CS-HL}=\sum_{k=1}^{K}w_{k}^{*}L_{k},\;\sum_{k=1}^{K}w_{k}^{*}=1,\;w_{k}^{*}\geq 0.$$

CS-HL affects only the training objective. During inference, no loss branch or weight-selection module is required. The final retrieval descriptor is obtained by concatenating the stage-wise features $f_{1},f_{2},f_{3}$, and $f_{4}$, so that fine-grained local cues and high-level identity semantics are jointly retained for baggage retrieval.

## 4. Experiments and Results

This section evaluates the proposed framework from three aspects. First, experiments on the self-collected BaggageID dataset are used to analyze backbone selection and to compare CaTFormer with reproducible open-source ReID models under the same baggage retrieval protocol. Second, experiments on the public MVB benchmark are used to position the proposed method among existing baggage ReID studies. Third, ablation studies and supplementary experiments are conducted to analyze component effectiveness, multi-level feature utilization, and performance on other public ReID datasets.

### 4.1. Datasets

**BaggageID.** The self-collected BaggageID dataset was obtained from the baggage handling system of a major airport in China. The system is equipped with multiple cameras, and 12 non-overlapping views were selected to capture baggage images along predefined transportation routes. Frames were extracted from surveillance videos, covering diverse viewpoints, backgrounds, illumination conditions, and motion states in real baggage handling scenarios. BaggageID contains 254 baggage identities and 17,613 images. Following airport business protocols, baggage items were categorized into four types, namely, hard case, soft case, framed, and empty frame. Colors were grouped into eight categories, and each baggage item was annotated with a color label. For evaluation, 177 baggage identities with 12,419 images were used for training, and the remaining 77 identities with 5194 images were used for testing.

BaggageID reflects a structured transportation surveillance scenario, where camera identifiers and simple baggage attributes can be associated with visual images. The textual attribute annotations were provided by a professional data annotation service following predefined labeling guidelines. Annotators were trained using unified attribute definitions, and cross-checking and manual verification were conducted to reduce subjective bias and labeling inconsistency. All data collection and annotation procedures complied with relevant airport data usage regulations and involved no personally identifiable information.

**MVB.** The Multi-View Baggage (MVB) dataset [28] is a public baggage ReID benchmark. It contains 4519 baggage identities and 22,660 annotated images, with a predefined split of 4019 identities for training and 500 identities for validation. MVB is constructed from two acquisition scenarios, namely, checkpoint and baggage handling system, and includes seven camera views in total. This cross-scenario and multi-view setting introduces appearance variations caused by viewpoint, background, and acquisition condition changes. MVB is used in this study to compare CaTFormer with existing baggage ReID methods reported in the literature.

**Public ReID datasets.** Additional experiments are conducted on Market1501 [33], VehicleID [34], and VeRi-776 [35] as supplementary evaluation beyond baggage images. Market1501 contains 32,668 images of 1501 pedestrians captured by six cameras. VehicleID includes 221,567 images of 26,328 vehicles collected from multiple city cameras. VeRi-776 comprises 49,357 images of 776 vehicles captured by 20 cameras, with annotations for vehicle type and color.

The statistics of the datasets used in this study are summarized in Table 1.

### 4.2. Implementation Details

All experiments were conducted in a deep learning environment based on PyTorch 1.13.1 and CUDA 11.4.3, with training and inference performed on an NVIDIA Tesla T4 GPU (NVIDIA Corporation, Santa Clara, CA, USA). The Swin transformer was initialized with weights pretrained on ImageNet-22K and further fine-tuned on ImageNet-1K. The embedding dimensionality used in Equation (1) was set to D=96, consistent with the default hidden dimension of the adopted Swin transformer backbone.

All input images were resized to 224×224. Data augmentation included random horizontal flipping, padding and random cropping, and random erasing [36]. We used stochastic gradient descent with a momentum of 0.9, a weight decay of $1\times 10^{-4}$, and a bias learning-rate factor of 2. The initial learning rate was 0.008, with linear warm-up during the first 5 epochs and a decay factor of 0.1 at epochs 40 and 70. Each model was trained for 120 epochs.

To construct identity-balanced mini-batches, each mini-batch contained *P* identities and *K* images per identity, giving a batch size of P×K. We set P=16 and K=4, resulting in a batch size of 64.

Performance was evaluated using the standard ReID retrieval protocol. The reported metrics were mean average precision (mAP), Rank-1, and Rank-5.

In addition to the general training settings, CS-HL requires the definition of candidate stage-weight vectors. The semantic-budget set and auxiliary-allocation pattern set were defined as

$$\begin{array}{c} B=\{0.55,0.60,0.65,0.70\},\\ P=\{\left(\frac{1}{3},\frac{1}{3},\frac{1}{3}\right),\left(0.50,0.25,0.25\right),\left(0.60,0.20,0.20\right),\\ \;(0.25,0.50,0.25),(0.25,0.25,0.50)\}. \end{array}$$

These settings yield 20 coarse semantic-dominant candidate weight vectors according to Equation (13). Candidate selection is separated from final evaluation. For each dataset, the official training identities are partitioned into an optimization subset and an identity-disjoint held-out validation subset. All coarse candidates are screened under the same short-training schedule using only this training-derived validation subset, and the leading candidates are retained according to validation mAP. A local refinement candidate set is then generated around the retained candidates while maintaining the semantic-dominant constraint $w_{4}>0.5$, and the refined candidates are screened on the same held-out validation subset. The resulting stage-weight vector is frozen before the final model is trained for 120 epochs using the full official training set. The official query and gallery sets are used only once for the final evaluation of the frozen selection.

To assess the run-to-run variability of the full CaTFormer configurations on BaggageID and MVB, the model was independently trained for 120 epochs with seeds 1234, 2024, and 3407. For each dataset, the fixed stage-weight vector, all remaining training settings, and the official evaluation protocol were kept identical across the three runs.

### 4.3. Backbone Selection on BaggageID

Before evaluating the full CaTFormer framework, we compare representative CNN, plain transformer, and hierarchical transformer backbones, including ResNet-50, ViT-B/16, and Swin-B, on the self-collected BaggageID dataset. This experiment uses the same basic ReID training setting without the proposed prior-embedding modules or CS-HL, and is designed only to justify the choice of visual backbone. The backbone-selection results are summarized in Table 2.

The results show that transformer-based backbones provide stronger retrieval performance than the CNN backbone on BaggageID under the basic ReID setting. Among the compared backbones, Swin-B achieves the highest mAP and Rank-1 accuracy, indicating that its hierarchical representation is suitable as the visual backbone for subsequent CaTFormer experiments. Therefore, Swin-B is adopted as the visual backbone in the following experiments.

### 4.4. Comparison with Representative ReID Methods on BaggageID

We compare CaTFormer with representative open-source ReID models on BaggageID under the same training and evaluation protocol. This comparison evaluates whether the proposed baggage-oriented prior-guided framework is effective under the self-collected baggage surveillance setting.

The results on BaggageID show that CaTFormer achieves better retrieval accuracy than the compared open-source ReID models under the same baggage evaluation protocol. The full model uses the frozen CS-HL vector [0.24, 0.08, 0.08, 0.60] together with the prior-guided fusion branch. These results suggest that structured camera and attribute priors, when coordinated with hierarchical visual supervision, are useful for baggage ReID scenarios characterized by high visual similarity and weak local identity cues.

For the seed-1234 run reported in Table 3, the final global coefficients of the three embedding branches were $\lambda_{cid}=0.4812$, $\lambda_{clr}=0.3522$, and $\lambda_{typ}=0.1666$, respectively. These values indicate that camera information received the largest global weight in this run.

**Figure 3:**
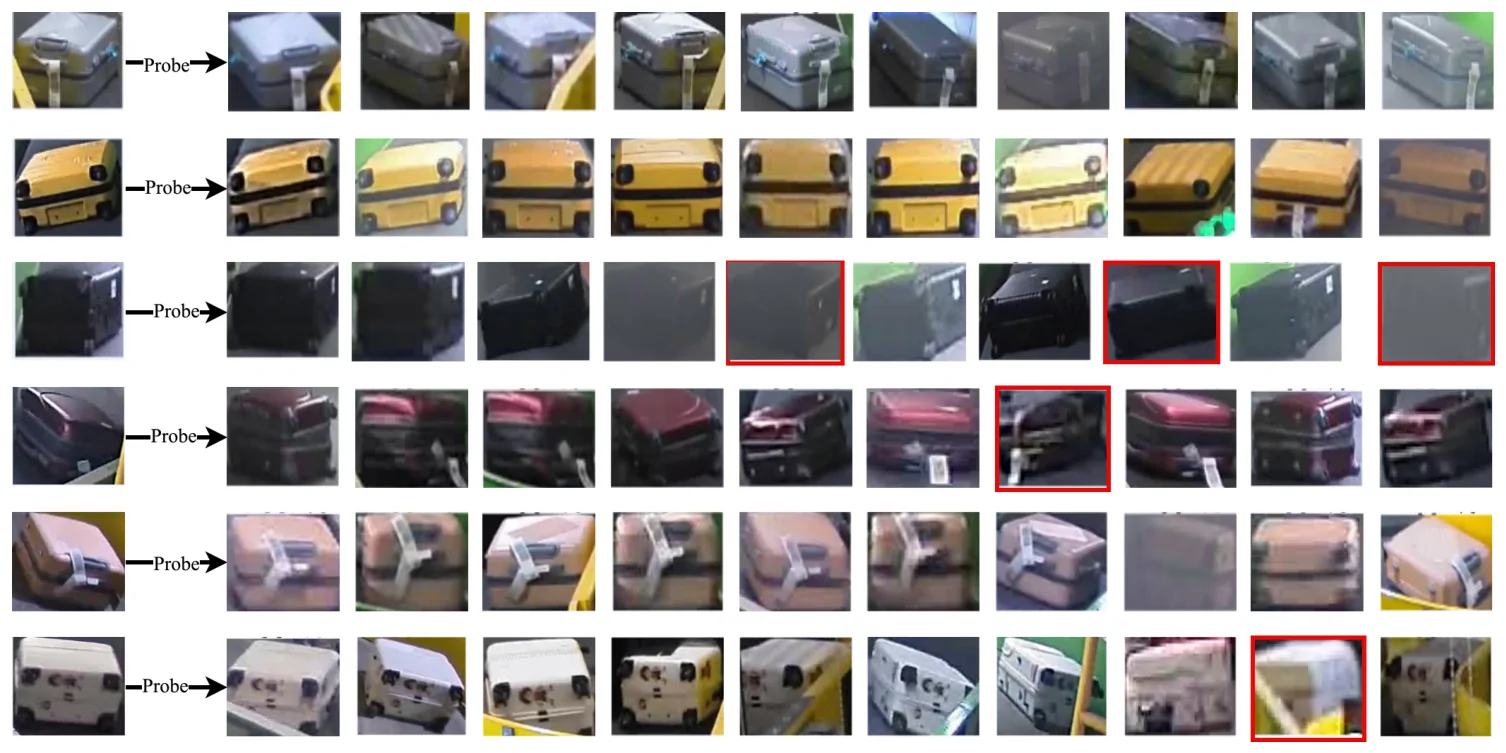
Visual example of baggage re-identification performance. Red boxes indicate incorrect retrievals.

Figure 3 provides a qualitative retrieval example on BaggageID. The retrieved images ranked near the query generally share the same baggage identity, while the red boxes mark incorrect retrieval results. This example complements the quantitative results in Table 3 by showing that the proposed framework can place visually similar positive samples in the top-ranked retrieval positions, although failure cases may still occur under high appearance similarity.

### 4.5. Comparison with Existing Baggage ReID Methods on MVB

We further evaluate CaTFormer on the public MVB benchmark to compare it with existing baggage ReID methods. The reported results of existing methods are collected from their corresponding MVB evaluations, and CaTFormer is evaluated under the MVB protocol.

To construct the textual-attribute embedding on MVB, color labels are annotated using the same eight-category definition as BaggageID. After coarse screening and local candidate refinement, the CS-HL stage-weight vector selected on MVB is [0.20, 0.09, 0.09, 0.62], and this vector is fixed during final training. The learned global AMEF branch coefficients on MVB are $\lambda_{cid}=0.273$, $\lambda_{clr}=0.467$, and $\lambda_{typ}=0.260$, indicating that color receives the largest global prior weight in this experiment.

The comparison results are summarized in Table 4.

As shown in Table 4, CaTFormer achieves 87.0% mAP and 87.6% Rank-1 accuracy on MVB. This evaluation extends the assessment of the proposed framework from the self-collected BaggageID dataset to an established public baggage ReID benchmark, where CaTFormer delivers competitive retrieval performance under a different data setting.

### 4.6. Ablation Studies

**Figure 4:**
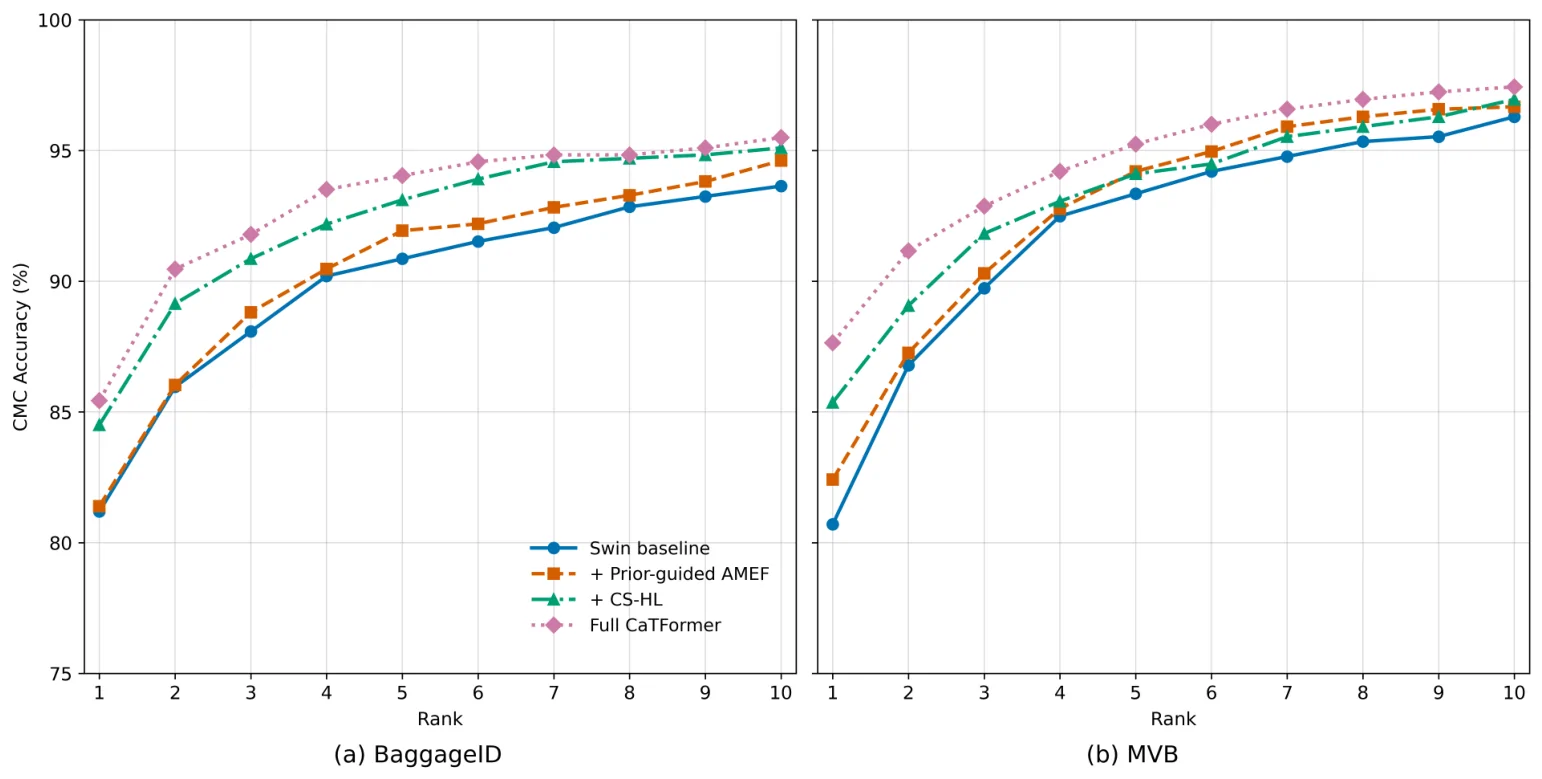
CMC curves for the ablation variants on the two datasets.

**Component Effectiveness.** This subsection evaluates the contribution of the main components in CaTFormer, including the prior-guided AMEF branch and CS-HL. The prior-guided AMEF branch refers to the structured-prior pathway that incorporates camera, type, and color embeddings and fuses them through AMEF. CS-HL is evaluated as the hierarchical supervision strategy for multi-level Swin features. Table 5 presents the mAP, Rank-1, and Rank-5 results of the ablation variants on BaggageID and MVB. Figure 4 further shows the CMC performance of the available model variants over ranks 1–10 on the two datasets.

As shown in Table 5, the prior-guided AMEF branch improves the baseline mAP on BaggageID from 55.9% to 57.9%, indicating that structured camera and attribute priors provide useful complementary cues. CS-HL further improves the baseline to 58.9% mAP and 84.5% Rank-1 by introducing hierarchical supervision. When both components are combined, the full CaTFormer achieves the best BaggageID performance, with 62.0% mAP, 85.4% Rank-1, and 94.0% Rank-5.

On MVB, both the prior-guided AMEF branch and CS-HL improve the baseline performance. When the two components are combined, the full CaTFormer achieves the best results among the ablated variants, with 87.0% mAP, 87.6% Rank-1, and 95.2% Rank-5. These results suggest that the proposed components are also effective on the MVB dataset.

To further characterize the run-to-run variability of the full CaTFormer configurations, we repeated the experiments on BaggageID and MVB using seeds 1234, 2024, and 3407. The BaggageID and MVB configurations achieved 62.2±0.3% and 85.8±1.1% mAP, respectively. The corresponding Rank-1 and Rank-5 results were 85.8±0.3% and 94.5±0.4% on BaggageID, and 86.4±0.9% and 95.1±0.3% on MVB (mean ± sample standard deviation). These repeated-run results complement the single-run evaluations by making the observed variability of the full CaTFormer explicit.

**Multi-Level Feature Fusion Strategy.** During feature representation, the outputs from different Swin stages are directly concatenated along the channel dimension to construct the final retrieval representation, without introducing an additional parameterized fusion module. This design keeps the representation simple and avoids extra fusion parameters. To examine whether explicit multi-level feature fusion is necessary, we compare the proposed direct-concatenation strategy with representative fusion modules, including feature pyramid network (FPN) [42], bidirectional FPN (BiFPN) [43], and attentional feature fusion (AFF) [44]. The comparison results are summarized in Table 6.

As shown in Table 6, FPN and BiFPN do not improve the Swin baseline, whereas AFF provides a moderate mAP gain. However, the concatenation-based strategy with CS-HL achieves the best overall result, reaching 58.9% mAP and 84.5% Rank-1. Moreover, adding explicit fusion modules on top of CS-HL does not further improve performance.

These results support the effectiveness of the concatenation-based representation strategy. With hierarchical supervision provided by CS-HL, multi-level Swin features can be exploited without requiring additional parameterized fusion modules.

### 4.7. Computational Cost Analysis

To quantify the computational characteristics of the proposed configurations, Table 7 reports the parameter count, FLOPs, and single-image inference latency of the locally implemented models on BaggageID under a unified profiling protocol. Because the stage-wise CS-HL heads are used only as auxiliary supervision during training and are removed after optimization, the reported values correspond to the retrieval network used for evaluation.

As shown in Table 7, the Swin baseline has 87.7767 M parameters and a computational cost of 30.3854 G FLOPs per image, with a model-only inference latency of 28.96±0.18 ms/image. Adding the prior-guided AMEF branch increases the parameter count by 0.0369 M, while the FLOPs remain nearly unchanged. The mean inference latency increases by 0.66 ms/image (2.27%). These results indicate that the prior-guided AMEF branch adds a small increase in model size and inference latency relative to the Swin baseline under the BaggageID setting.

### 4.8. Supplementary Evaluation on Public ReID Benchmarks

Finally, we report supplementary evaluation on public person and vehicle ReID benchmarks beyond baggage images. The full CaTFormer framework is evaluated on VeRi-776, where camera identifiers and vehicle attributes are available. When the required priors are incomplete, as in Market1501 and VehicleID, the reduced Swin-CS-HL variant is evaluated using the same stage-weight vector as in the BaggageID experiment. The supplementary results are summarized in Table 8.

As shown in Table 8, CaTFormer achieves a competitive Rank-1 accuracy on VeRi-776, while CLIP-ReID obtains the highest mAP and MBR_4B obtains the highest Rank-1 accuracy on this dataset. On Market1501 and VehicleID, the reduced Swin-CS-HL variant produces competitive but not leading results compared with representative ReID methods. These results indicate that hierarchical supervision remains useful in other ReID settings and can provide competitive retrieval representations beyond baggage images.

## 5. Conclusions

In this study, we propose CaTFormer, a baggage-oriented transformer framework for structured transportation surveillance scenarios. The framework incorporates camera-aware and textual-attribute embeddings into Swin-based visual representation learning through the AMEF module, which coordinates camera, color, and type priors before injecting them into visual tokens. In addition, CS-HL supervises multi-level Swin features using a candidate-selected semantic-dominant weight vector, so that shallow fine-grained cues and deeper semantic representations can be jointly optimized during training.

Experiments on the self-collected BaggageID dataset show that CaTFormer achieves the highest mAP among the compared open-source ReID models under the same evaluation protocol, reaching 62.0% mAP, 85.4% Rank-1 accuracy, and 94.0% Rank-5 accuracy. On the public MVB benchmark, CaTFormer obtains 87.0% mAP and 87.6% Rank-1 accuracy, showing competitive performance in the comparison with existing baggage ReID methods. The ablation results further show that the prior-guided AMEF branch and CS-HL both contribute to retrieval performance. In addition, the comparison of multi-level feature fusion strategies indicates that direct concatenation, when combined with hierarchical supervision, can exploit multi-stage Swin features without introducing additional parameterized fusion modules. Supplementary evaluation on public person and vehicle ReID datasets also suggests that hierarchical supervision remains useful beyond baggage images.

Despite these results, several aspects of the current framework require further investigation. Although AMEF adaptively coordinates camera, color, and type priors, the use of these priors still depends on their availability and annotation consistency in practical baggage handling systems. The current attribute space is also limited to basic color and type labels, and future work may incorporate automatic attribute extraction, open-vocabulary visual descriptions, or multimodal baggage descriptors to provide richer semantic cues. In addition, CS-HL currently relies on candidate screening to select the stage-weight vector; more adaptive or screening-efficient hierarchical weighting strategies should be investigated to reduce repeated screening while maintaining stable multi-level supervision. Broader validation across airports, camera layouts, and operational conditions is also needed to further assess its applicability in practical baggage management systems.

## Figures and Tables

**Table 1:** Statistics of datasets used for evaluation.

Dataset	Object	ID	Image	Camera	Color	Type
BaggageID	Baggage	254	17,613	12	8	4
MVB	Baggage	4519	22,660	7	–	4
VeRi-776	Vehicle	776	49,357	20	10	9
VehicleID	Vehicle	26,328	221,567	–	–	–
Market1501	Person	1501	32,668	6	–	–

**Table 2:** Backbone selection on the self-collected BaggageID dataset.

Backbone	mAP	Rank-1	Rank-5
ResNet-50	40.2%	68.7%	84.2%
ViT-B/16	54.1%	79.6%	**91.4%**
Swin-B	**55.9%**	**81.3%**	90.9%

*Note*: The best result in each column is shown in bold.

**Table 3:** Comparison with representative ReID methods on BaggageID under the same protocol.

Method	mAP	Rank-1	Rank-5
RRID [20]	46.9%	71.4%	87.8%
CAL [37]	50.7%	76.3%	88.7%
FastReID (r50-ibn) [38]	52.8%	80.5%	91.7%
FastReID (sbs_r50_ibn) [38]	54.6%	82.3%	90.9%
TransReID [3]	58.3%	82.9%	91.8%
MBR_4B [39]	57.5%	82.8%	91.6%
CLIP-ReID [40]	53.7%	78.4%	89.5%
FusionReID [15]	57.4%	82.9%	93.0%
CaTFormer	**62.0%**	**85.4%**	**94.0%**

*Note*: The best result in each column is shown in bold.

**Table 4:** Comparison with existing baggage ReID methods on the public MVB benchmark.

Method	mAP	Rank-1
MSN [28]	51.8%	50.2%
Siamese Network [6]	63.1%	69.1%
LSDNN [8]	84.7%	82.4%
AMG [41]	82.5%	84.6%
SMBNet [7]	84.7%	82.8%
QuadNet [9]	86.1%	87.5%
CDR-CARNet [10]	87.0%	86.1%
RGViT [30]	89.6%	88.7%
CaTFormer	87.0%	87.6%

**Table 5:** Ablation study of model components on BaggageID and MVB.

Model Variant	BaggageID			MVB		
	mAP	Rank-1	Rank-5	mAP	Rank-1	Rank-5
Swin baseline	55.9%	81.3%	90.9%	78.6%	80.7%	93.3%
Swin + prior-guided AMEF	57.9%	81.1%	91.9%	81.1%	82.4%	94.2%
Swin + CS-HL	58.9%	84.5%	93.1%	85.2%	85.4%	94.1%
Full CaTFormer	**62.0%**	**85.4%**	**94.0%**	**87.0%**	**87.6%**	**95.2%**

*Note*: The best result in each column is shown in bold.

**Table 6:** Comparison of hierarchical supervision and feature fusion strategies on BaggageID.

Method	mAP	Rank-1	Rank-5
Swin	55.9%	81.3%	90.9%
Swin + FPN	54.7%	80.7%	90.2%
Swin + BiFPN	54.0%	82.0%	90.9%
Swin + AFF	57.2%	82.0%	91.7%
Swin + FPN + CS-HL	55.6%	79.6%	90.7%
Swin + BiFPN + CS-HL	49.8%	77.2%	90.3%
Swin + AFF + CS-HL	48.9%	76.3%	89.2%
Swin + CS-HL	**58.9%**	**84.5%**	**93.1%**

*Note*: The best result in each column is shown in bold.

**Table 7:** Comparison of inference-time model complexity on BaggageID.

Model	Params (M)	FLOPs (G)	Latency (ms/Image)
Swin baseline	87.7767	30.3854	28.96±0.18
Swin + prior-guided AMEF	87.8135	30.3855	29.62±0.17

**Table 8:** Supplementary comparison on public ReID datasets.

Method	Market1501		VeRi-776		VehicleID	
	mAP	Rank-1	mAP	Rank-1	mAP	Rank-1
RRID [20]	88.9%	95.2%	77.4%	90.2%	86.9%	80.3%
CAL [37]	89.5%	95.5%	74.3%	95.4%	87.8%	82.5%
FastReID [38]	87.5%	95.1%	81.9%	97.0%	85.7%	79.8%
TransReID [3]	88.3%	95.4%	81.0%	97.0%	87.6%	81.3%
MBR_4B [39]	81.7%	86.3%	83.3%	**97.6%**	**91.8%**	**87.3%**
CLIP-ReID [40]	90.5%	95.4%	**84.5%**	97.3%	90.6%	85.3%
FusionReID [15]	**91.7%**	**96.3%**	81.4%	96.3%	88.9%	82.6%
Swin-CS-HL	88.2%	94.9%	81.0%	96.4%	89.1%	83.1%
CaTFormer	–	–	82.4%	97.5%	–	–

*Note*: The best result in each column is shown in bold.

## Data Availability

The public datasets used in this study, namely, Market1501, VeRi-776, VehicleID and MVB, can be accessed from the references [28,33,34,35], respectively. The private BaggageID dataset was collected from a real-world airport baggage handling system and is subject to data usage regulations and confidentiality constraints; therefore, it cannot be publicly released or shared with third parties.
